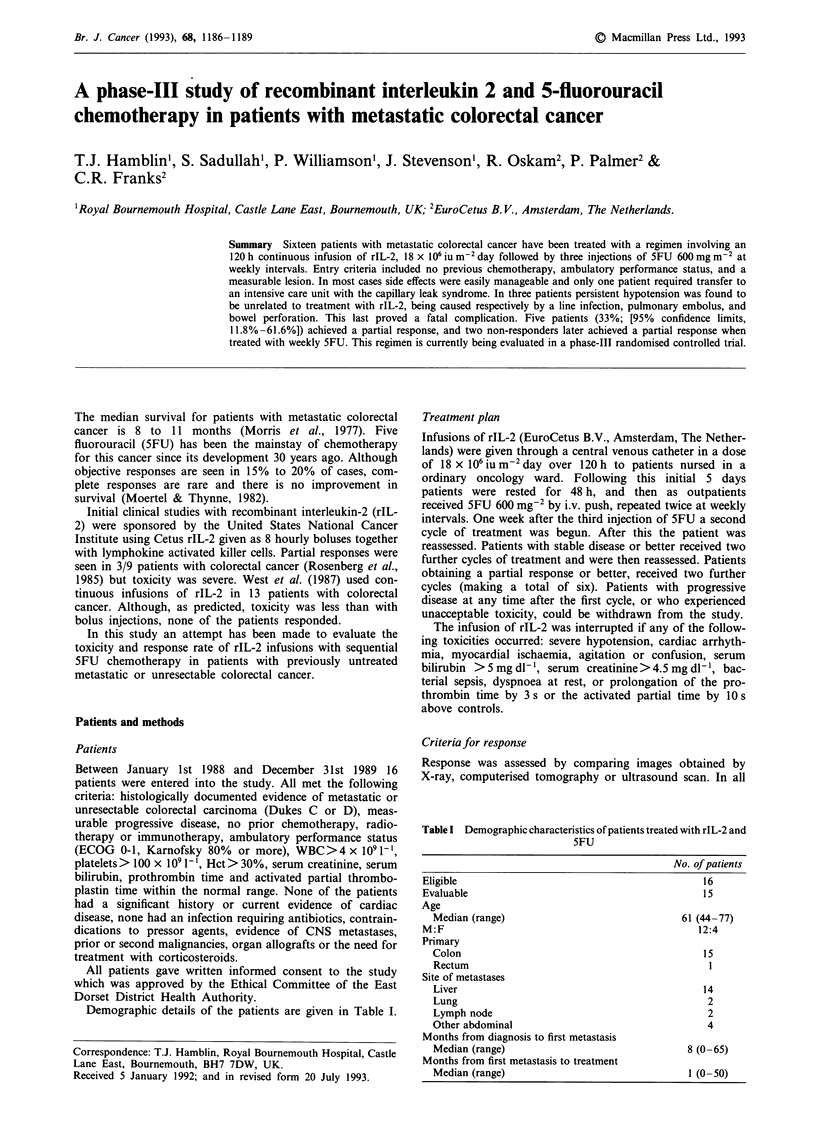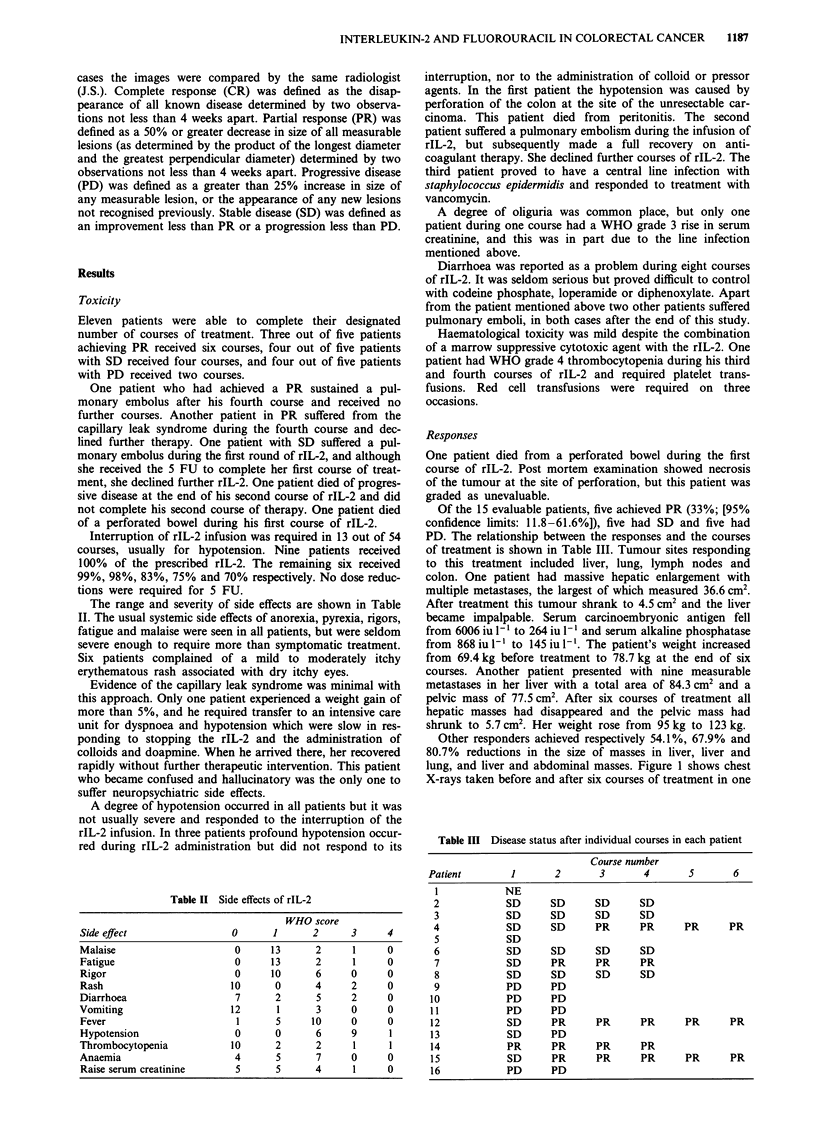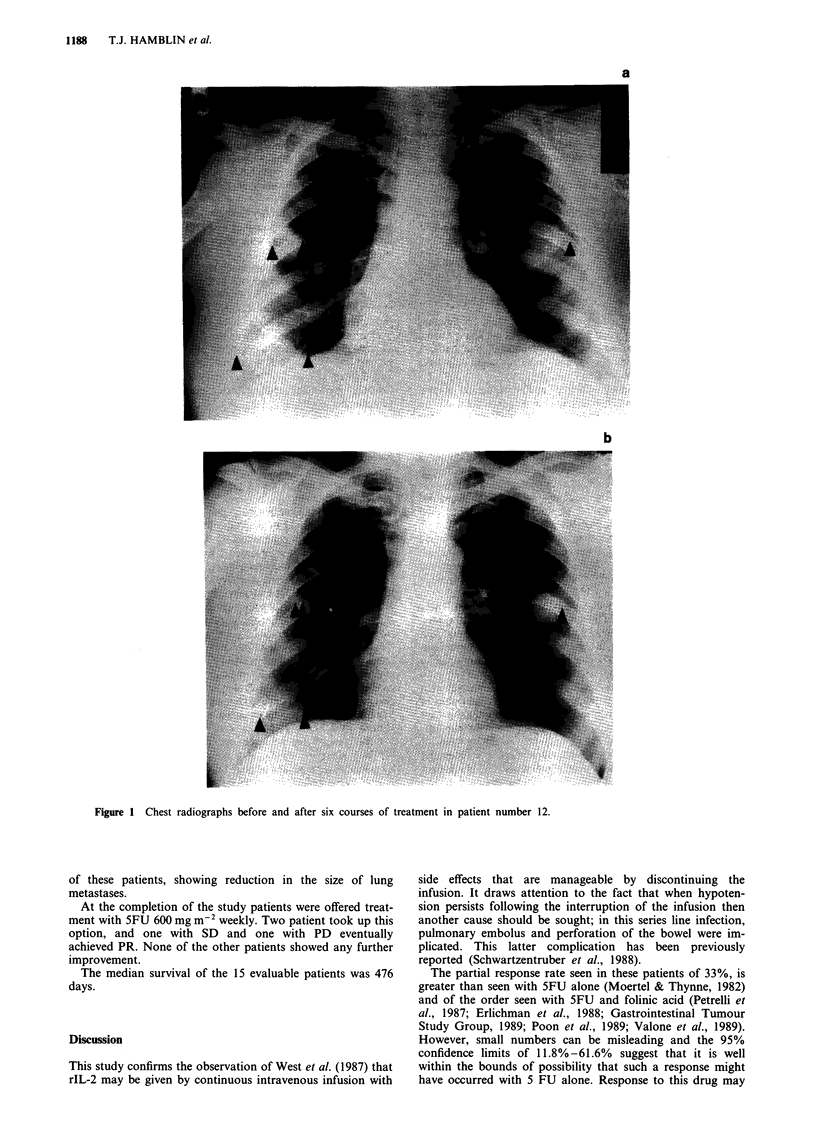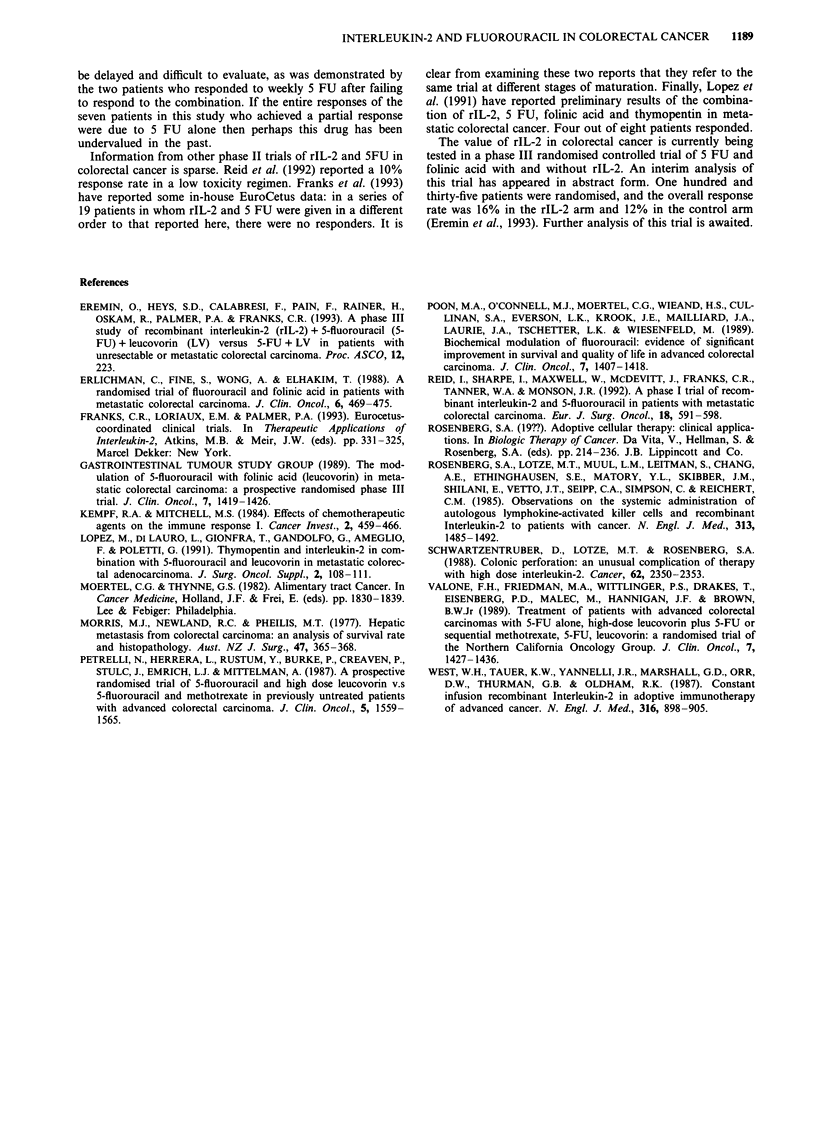# A phase-III study of recombinant interleukin 2 and 5-fluorouracil chemotherapy in patients with metastatic colorectal cancer.

**DOI:** 10.1038/bjc.1993.501

**Published:** 1993-12

**Authors:** T. J. Hamblin, S. Sadullah, P. Williamson, J. Stevenson, R. Oskam, P. Palmer, C. R. Franks

**Affiliations:** Royal Bournemouth Hospital, UK.

## Abstract

**Images:**


Br. J. Cancer (1993), 68, 1186–1189 © Macmillan Press Ltd., 1993

### A phase-III study of recombinant interleukin 2 and 5-fluorouracil chemotherapy in patients with metastatic colorectal cancer

T.J. Hamblin[1], S. Sadullah[1], P. Williamson[1], J. Stevenson[1], R. Oskam[2], P. Palmer[2] & C.R. Franks[2]

[1]*Royal Bournemouth Hospital, Castle Lane East, Bournemouth, UK;* [2]*EuroCetus B.V., Amsterdam, The Netherlands.*

**Summary** Sixteen patients with metastatic colorectal cancer have been treated with a regimen involving an 120 h continuous infusion of rIL-2, $18 \times 10^6$ iu $m^{-2}$ day followed by three injections of 5FU 600 mg $m^{-2}$ at weekly intervals. Entry criteria included no previous chemotherapy, ambulatory performance status, and a measurable lesion. In most cases side effects were easily manageable and only one patient required transfer to an intensive care unit with the capillary leak syndrome. In three patients persistent hypotension was found to be unrelated to treatment with rIL-2, being caused respectively by a line infection, pulmonary embolus, and bowel perforation. This last proved a fatal complication. Five patients (33%; [95% confidence limits, 11.8%–61.6%]) achieved a partial response, and two non-responders later achieved a partial response when treated with weekly 5FU. This regimen is currently being evaluated in a phase-III randomised controlled trial.

The median survival for patients with metastatic colorectal cancer is 8 to 11 months (Morris *et al.*, 1977). Five fluorouracil (5FU) has been the mainstay of chemotherapy for this cancer since its development 30 years ago. Although objective responses are seen in 15% to 20% of cases, complete responses are rare and there is no improvement in survival (Moertel & Thynne, 1982).

Initial clinical studies with recombinant interleukin-2 (rIL-2) were sponsored by the United States National Cancer Institute using Cetus rIL-2 given as 8 hourly boluses together with lymphokine activated killer cells. Partial responses were seen in 3/9 patients with colorectal cancer (Rosenberg *et al.*, 1985) but toxicity was severe. West *et al.* (1987) used continuous infusions of rIL-2 in 13 patients with colorectal cancer. Although, as predicted, toxicity was less than with bolus injections, none of the patients responded.

In this study an attempt has been made to evaluate the toxicity and response rate of rIL-2 infusions with sequential 5FU chemotherapy in patients with previously untreated metastatic or unresectable colorectal cancer.

### Patients and methods

### *Patients*

Between January 1st 1988 and December 31st 1989 16 patients were entered into the study. All met the following criteria: histologically documented evidence of metastatic or unresectable colorectal carcinoma (Dukes C or D), measurable progressive disease, no prior chemotherapy, radiotherapy or immunotherapy, ambulatory performance status (ECOG 0-1, Karnofsky 80% or more), WBC $> 4 \times 10^9 l^{-1}$, platelets $> 100 \times 10^9 l^{-1}$, Hct > 30%, serum creatinine, serum bilirubin, prothrombin time and activated partial thromboplastin time within the normal range. None of the patients had a significant history or current evidence of cardiac disease, none had an infection requiring antibiotics, contraindications to pressor agents, evidence of CNS metastases, prior or second malignancies, organ allografts or the need for treatment with corticosteroids.

All patients gave written informed consent to the study which was approved by the Ethical Committee of the East Dorset District Health Authority.

Demographic details of the patients are given in Table I.

### *Treatment plan*

Infusions of rIL-2 (EuroCetus B.V., Amsterdam, The Netherlands) were given through a central venous catheter in a dose of $18 \times 10^6$ iu $m^{-2}$ day over 120 h to patients nursed in a ordinary oncology ward. Following this initial 5 days patients were rested for 48 h, and then as outpatients received 5FU 600 mg$^{-2}$ by i.v. push, repeated twice at weekly intervals. One week after the third injection of 5FU a second cycle of treatment was begun. After this the patient was reassessed. Patients with stable disease or better received two further cycles of treatment and were then reassessed. Patients obtaining a partial response or better, received two further cycles (making a total of six). Patients with progressive disease at any time after the first cycle, or who experienced unacceptable toxicity, could be withdrawn from the study.

The infusion of rIL-2 was interrupted if any of the following toxicities occurred: severe hypotension, cardiac arrhythmia, myocardial ischaemia, agitation or confusion, serum bilirubin $> 5$ mg $dl^{-1}$, serum creatinine $> 4.5$ mg $dl^{-1}$, bacterial sepsis, dyspnoea at rest, or prolongation of the prothrombin time by 3 s or the activated partial time by 10 s above controls.

### *Criteria for response*

Response was assessed by comparing images obtained by X-ray, computerised tomography or ultrasound scan. In all

**Table I** Demographic characteristics of patients treated with rIL-2 and 5FU

| | *No. of patients* |
|---|---|
| Eligible | 16 |
| Evaluable | 15 |
| Age | |
| Median (range) | 61 (44–77) |
| M:F | 12:4 |
| Primary | |
| Colon | 15 |
| Rectum | 1 |
| Site of metastases | |
| Liver | 14 |
| Lung | 2 |
| Lymph node | 2 |
| Other abdominal | 4 |
| Months from diagnosis to first metastasis | |
| Median (range) | 8 (0–65) |
| Months from first metastasis to treatment | |
| Median (range) | 1 (0–50) |

Correspondence: T.J. Hamblin, Royal Bournemouth Hospital, Castle Lane East, Bournemouth, BH7 7DW, UK.
Received 5 January 1992; and in revised form 20 July 1993.

cases the images were compared by the same radiologist (J.S.). Complete response (CR) was defined as the disappearance of all known disease determined by two observations not less than 4 weeks apart. Partial response (PR) was defined as a 50% or greater decrease in size of all measurable lesions (as determined by the product of the longest diameter and the greatest perpendicular diameter) determined by two observations not less than 4 weeks apart. Progressive disease (PD) was defined as a greater than 25% increase in size of any measurable lesion, or the appearance of any new lesions not recognised previously. Stable disease (SD) was defined as an improvement less than PR or a progression less than PD.

### Results

### *Toxicity*

Eleven patients were able to complete their designated number of courses of treatment. Three out of five patients achieving PR received six courses, four out of five patients with SD received four courses, and four out of five patients with PD received two courses.

One patient who had achieved a PR sustained a pulmonary embolus after his fourth course and received no further courses. Another patient in PR suffered from the capillary leak syndrome during the fourth course and declined further therapy. One patient with SD suffered a pulmonary embolus during the first round of rIL-2, and although she received the 5 FU to complete her first course of treatment, she declined further rIL-2. One patient died of progressive disease at the end of his second course of rIL-2 and did not complete his second course of therapy. One patient died of a perforated bowel during his first course of rIL-2.

Interruption of rIL-2 infusion was required in 13 out of 54 courses, usually for hypotension. Nine patients received 100% of the prescribed rIL-2. The remaining six received 99%, 98%, 83%, 75% and 70% respectively. No dose reductions were required for 5 FU.

The range and severity of side effects are shown in Table II. The usual systemic side effects of anorexia, pyrexia, rigors, fatigue and malaise were seen in all patients, but were seldom severe enough to require more than symptomatic treatment. Six patients complained of a mild to moderately itchy erythematous rash associated with dry itchy eyes.

Evidence of the capillary leak syndrome was minimal with this approach. Only one patient experienced a weight gain of more than 5%, and he required transfer to an intensive care unit for dyspnoea and hypotension which were slow in responding to stopping the rIL-2 and the administration of colloids and doapmine. When he arrived there, her recovered rapidly without further therapeutic intervention. This patient who became confused and hallucinatory was the only one to suffer neuropsychiatric side effects.

A degree of hypotension occurred in all patients but it was not usually severe and responded to the interruption of the rIL-2 infusion. In three patients profound hypotension occurred during rIL-2 administration but did not respond to its interruption, nor to the administration of colloid or pressor agents. In the first patient the hypotension was caused by perforation of the colon at the site of the unresectable carcinoma. This patient died from peritonitis. The second patient suffered a pulmonary embolism during the infusion of rIL-2, but subsequently made a full recovery on anticoagulant therapy. She declined further courses of rIL-2. The third patient proved to have a central line infection with *staphylococcus epidermidis* and responded to treatment with vancomycin.

A degree of oliguria was common place, but only one patient during one course had a WHO grade 3 rise in serum creatinine, and this was in part due to the line infection mentioned above.

Diarrhoea was reported as a problem during eight courses of rIL-2. It was seldom serious but proved difficult to control with codeine phosphate, loperamide or diphenoxylate. Apart from the patient mentioned above two other patients suffered pulmonary emboli, in both cases after the end of this study.

Haematological toxicity was mild despite the combination of a marrow suppressive cytotoxic agent with the rIL-2. One patient had WHO grade 4 thrombocytopenia during his third and fourth courses of rIL-2 and required platelet transfusions. Red cell transfusions were required on three occasions.

### *Responses*

One patient died from a perforated bowel during the first course of rIL-2. Post mortem examination showed necrosis of the tumour at the site of perforation, but this patient was graded as unevaluable.

Of the 15 evaluable patients, five achieved PR (33%; [95% confidence limits: 11.8–61.6%]), five had SD and five had PD. The relationship between the responses and the courses of treatment is shown in Table III. Tumour sites responding to this treatment included liver, lung, lymph nodes and colon. One patient had massive hepatic enlargement with multiple metastases, the largest of which measured 36.6 $cm^2$. After treatment this tumour shrank to 4.5 $cm^2$ and the liver became impalpable. Serum carcinoembryonic antigen fell from 6006 iu $l^{-1}$ to 264 iu $l^{-1}$ and serum alkaline phosphatase from 868 iu $l^{-1}$ to 145 iu $l^{-1}$. The patient's weight increased from 69.4 kg before treatment to 78.7 kg at the end of six courses. Another patient presented with nine measurable metastases in her liver with a total area of 84.3 $cm^2$ and a pelvic mass of 77.5 $cm^2$. After six courses of treatment all hepatic masses had disappeared and the pelvic mass had shrunk to 5.7 $cm^2$. Her weight rose from 95 kg to 123 kg.

Other responders achieved respectively 54.1%, 67.9% and 80.7% reductions in the size of masses in liver, liver and lung, and liver and abdominal masses. Figure 1 shows chest X-rays taken before and after six courses of treatment in one

**Table II** Side effects of rIL-2

| Side effect | WHO score 0 | 1 | 2 | 3 | 4 |
|---|---|---|---|---|---|
| Malaise | 0 | 13 | 2 | 1 | 0 |
| Fatigue | 0 | 13 | 2 | 1 | 0 |
| Rigor | 0 | 10 | 6 | 0 | 0 |
| Rash | 10 | 0 | 4 | 2 | 0 |
| Diarrhoea | 7 | 2 | 5 | 2 | 0 |
| Vomiting | 12 | 1 | 3 | 0 | 0 |
| Fever | 1 | 5 | 10 | 0 | 0 |
| Hypotension | 0 | 0 | 6 | 9 | 1 |
| Thrombocytopenia | 10 | 2 | 2 | 1 | 1 |
| Anaemia | 4 | 5 | 7 | 0 | 0 |
| Raise serum creatinine | 5 | 5 | 4 | 1 | 0 |

**Table III** Disease status after individual courses in each patient

| Patient | Course number 1 | 2 | 3 | 4 | 5 | 6 |
|---|---|---|---|---|---|---|
| 1 | NE | | | | | |
| 2 | SD | SD | SD | SD | | |
| 3 | SD | SD | SD | SD | | |
| 4 | SD | SD | PR | PR | PR | PR |
| 5 | SD | | | | | |
| 6 | SD | SD | SD | SD | | |
| 7 | SD | PR | PR | PR | | |
| 8 | SD | SD | SD | SD | | |
| 9 | PD | PD | | | | |
| 10 | PD | PD | | | | |
| 11 | PD | PD | | | | |
| 12 | SD | PR | PR | PR | PR | PR |
| 13 | SD | PD | | | | |
| 14 | PR | PR | PR | PR | | |
| 15 | SD | PR | PR | PR | PR | PR |
| 16 | PD | PD | | | | |

**Figure 1** Chest radiographs before and after six courses of treatment in patient number 12.

of these patients, showing reduction in the size of lung metastases.

At the completion of the study patients were offered treatment with 5FU 600 mg $m^{-2}$ weekly. Two patient took up this option, and one with SD and one with PD eventually achieved PR. None of the other patients showed any further improvement.

The median survival of the 15 evaluable patients was 476 days.

### Discussion

This study confirms the observation of West *et al.* (1987) that rIL-2 may be given by continuous intravenous infusion with side effects that are manageable by discontinuing the infusion. It draws attention to the fact that when hypotension persists following the interruption of the infusion then another cause should be sought; in this series line infection, pulmonary embolus and perforation of the bowel were implicated. This latter complication has been previously reported (Schwartzentruber *et al.*, 1988).

The partial response rate seen in these patients of 33%, is greater than seen with 5FU alone (Moertel & Thynne, 1982) and of the order seen with 5FU and folinic acid (Petrelli *et al.*, 1987; Erlichman *et al.*, 1988; Gastrointestinal Tumour Study Group, 1989; Poon *et al.*, 1989; Valone *et al.*, 1989). However, small numbers can be misleading and the 95% confidence limits of 11.8%–61.6% suggest that it is well within the bounds of possibility that such a response might have occurred with 5 FU alone. Response to this drug may be delayed and difficult to evaluate, as was demonstrated by the two patients who responded to weekly 5 FU after failing to respond to the combination. If the entire responses of the seven patients in this study who achieved a partial response were due to 5 FU alone then perhaps this drug has been undervalued in the past.

Information from other phase II trials of rIL-2 and 5FU in colorectal cancer is sparse. Reid *et al.* (1992) reported a 10% response rate in a low toxicity regimen. Franks *et al.* (1993) have reported some in-house EuroCetus data: in a series of 19 patients in whom rIL-2 and 5 FU were given in a different order to that reported here, there were no responders. It is clear from examining these two reports that they refer to the same trial at different stages of maturation. Finally, Lopez *et al.* (1991) have reported preliminary results of the combination of rIL-2, 5 FU, folinic acid and thymopentin in metastatic colorectal cancer. Four out of eight patients responded.

The value of rIL-2 in colorectal cancer is currently being tested in a phase III randomised controlled trial of 5 FU and folinic acid with and without rIL-2. An interim analysis of this trial has appeared in abstract form. One hundred and thirty-five patients were randomised, and the overall response rate was 16% in the rIL-2 arm and 12% in the control arm (Eremin *et al.*, 1993). Further analysis of this trial is awaited.

### References

EREMIN, O., HEYS, S.D., CALABRESI, F., PAIN, F., RAINER, H., OSKAM, R., PALMER, P.A. & FRANKS, C.R. (1993). A phase III study of recombinant interleukin-2 (rIL-2) + 5-fluorouracil (5-FU) + leucovorin (LV) versus 5-FU + LV in patients with unresectable or metastatic colorectal carcinoma. *Proc. ASCO*, **12**, 223.

ERLICHMAN, C., FINE, S., WONG, A. & ELHAKIM, T. (1988). A randomised trial of fluorouracil and folinic acid in patients with metastatic colorectal carcinoma. *J. Clin. Oncol.*, **6**, 469-475.

FRANKS, C.R., LORIAUX, E.M. & PALMER, P.A. (1993). Eurocetus-coordinated clinical trials. In *Therapeutic Applications of Interleukin-2*, Atkins, M.B. & Meir, J.W. (eds). pp. 331-325, Marcel Dekker: New York.

GASTROINTESTINAL TUMOUR STUDY GROUP (1989). The modulation of 5-fluorouracil with folinic acid (leucovorin) in metastatic colorectal carcinoma: a prospective randomised phase III trial. *J. Clin. Oncol.*, **7**, 1419-1426.

KEMPF, R.A. & MITCHELL, M.S. (1984). Effects of chemotherapeutic agents on the immune response I. *Cancer Invest.*, **2**, 459-466.

LOPEZ, M., DI LAURO, L., GIONFRA, T., GANDOLFO, G., AMEGLIO, F. & POLETTI, G. (1991). Thymopentin and interleukin-2 in combination with 5-fluorouracil and leucovorin in metastatic colorectal adenocarcinoma. *J. Surg. Oncol. Suppl.*, **2**, 108-111.

MOERTEL, C.G. & THYNNE, G.S. (1982). Alimentary tract Cancer. In *Cancer Medicine*, Holland, J.F. & Frei, E. (eds). pp. 1830-1839. Lee & Febiger: Philadelphia.

MORRIS, M.J., NEWLAND, R.C. & PHEILIS, M.T. (1977). Hepatic metastasis from colorectal carcinoma: an analysis of survival rate and histopathology. *Aust. NZ J. Surg.*, **47**, 365-368.

PETRELLI, N., HERRERA, L., RUSTUM, Y., BURKE, P., CREAVEN, P., STULC, J., EMRICH, L.J. & MITTELMAN, A. (1987). A prospective randomised trial of 5-fluorouracil and high dose leucovorin v.s 5-fluorouracil and methotrexate in previously untreated patients with advanced colorectal carcinoma. *J. Clin. Oncol.*, **5**, 1559-1565.

POON, M.A., O'CONNELL, M.J., MOERTEL, C.G., WIEAND, H.S., CULLINAN, S.A., EVERSON, L.K., KROOK, J.E., MAILLIARD, J.A., LAURIE, J.A., TSCHETTER, L.K. & WIESENFELD, M. (1989). Biochemical modulation of fluorouracil: evidence of significant improvement in survival and quality of life in advanced colorectal carcinoma. *J. Clin. Oncol.*, **7**, 1407-1418.

REID, I., SHARPE, I., MAXWELL, W., MCDEVITT, J., FRANKS, C.R., TANNER, W.A. & MONSON, J.R. (1992). A phase I trial of recombinant interleukin-2 and 5-fluorouracil in patients with metastatic colorectal carcinoma. *Eur. J. Surg. Oncol.*, **18**, 591-598.

ROSENBERG, S.A. (19??). Adoptive cellular therapy: clinical applications. In *Biologic Therapy of Cancer*. Da Vita, V., Hellman, S. & Rosenberg, S.A. (eds). pp. 214-236. J.B. Lippincott and Co.

ROSENBERG, S.A., LOTZE, M.T., MUUL, L.M., LEITMAN, S., CHANG, A.E., ETHINGHAUSEN, S.E., MATORY, Y.L., SKIBBER, J.M., SHILANI, E., VETTO, J.T., SEIPP, C.A., SIMPSON, C. & REICHERT, C.M. (1985). Observations on the systemic administration of autologous lymphokine-activated killer cells and recombinant Interleukin-2 to patients with cancer. *N. Engl. J. Med.*, **313**, 1485-1492.

SCHWARTZENTRUBER, D., LOTZE, M.T. & ROSENBERG, S.A. (1988). Colonic perforation: an unusual complication of therapy with high dose interleukin-2. *Cancer*, **62**, 2350-2353.

VALONE, F.H., FRIEDMAN, M.A., WITTLINGER, P.S., DRAKES, T., EISENBERG, P.D., MALEC, M., HANNIGAN, J.F. & BROWN, B.W.Jr (1989). Treatment of patients with advanced colorectal carcinomas with 5-FU alone, high-dose leucovorin plus 5-FU or sequential methotrexate, 5-FU, leucovorin: a randomised trial of the Northern California Oncology Group. *J. Clin. Oncol.*, **7**, 1427-1436.

WEST, W.H., TAUER, K.W., YANNELLI, J.R., MARSHALL, G.D., ORR, D.W., THURMAN, G.B. & OLDHAM, R.K. (1987). Constant infusion recombinant Interleukin-2 in adoptive immunotherapy of advanced cancer. *N. Engl. J. Med.*, **316**, 898-905.